# Proteome-Wide Serological Profiling Reveals Broad Elevation of EBV Immunity in Idiopathic Pulmonary Fibrosis

**DOI:** 10.3390/ijms27020783

**Published:** 2026-01-13

**Authors:** Yomani D. Sarathkumara, Kiara M. Knuckey, Viviana P. Lutzky, Penny L. Groves, Maxine E. Tan, Daniel C. Chambers, Carla Proietti, Denise L. Doolan, Simon H. Apte

**Affiliations:** 1 Institute for Molecular Bioscience, The University of Queensland, St. Lucia, QLD 4072, Australiac.proietti@uq.edu.au (C.P.); 2 Queensland Lung Transplant Service, The Prince Charles Hospital, Chermside, QLD 4032, Australiadaniel.chambers@health.qld.gov.au (D.C.C.); 3 Faculty of Health, Medicine and Behavioural Sciences, The University of Queensland, St. Lucia, QLD 4072, Australia

**Keywords:** idiopathic pulmonary fibrosis, Epstein–Barr virus, antibody, microarray

## Abstract

Idiopathic pulmonary fibrosis (IPF) is a progressive interstitial lung disease with uncertain etiology. Chronic viral infection, including Epstein–Barr virus (EBV), has been implicated as a potential driver of repetitive epithelial injury and dysregulated repair. We sought to evaluate and define the breadth versus specificity of EBV-directed humoral immunity in IPF. We performed proteome-scale serological profiling using an EBV protein microarray (202 proteins) representing all proteins expressed by the EBV proteome (type I and II) on plasma samples from 32 patients with confirmed IPF (87.5% male; mean age 60.9 years) and 15 healthy disease-free controls (40% male; mean age 57.9 years). Per-sample global EBV IgG means were higher in IPF than controls (Welch *p* = 0.005), and the difference persisted after sex adjustment (*p* = 0.012). Although no single antigen met a stringent FDR significance threshold, 10 EBV antigen-specific antibody responses showed nominal elevation in IPF, with 2 remaining nominally significant after sex adjustment and 5 additional antibody responses reaching significance only in linear regression models. Overall, these results support the concept that IPF is associated with a diffuse elevation of EBV-directed humoral responses rather than antigen-specific dominance, consistent with ongoing, low-level viral reactivation. The presence of an EBV-negative subgroup within the IPF cohort underscores etiological heterogeneity within IPF.

## 1. Introduction

Idiopathic pulmonary fibrosis (IPF) is a chronic, progressive, fibrotic interstitial lung disease of unknown cause, typically affecting older adults and characterized by inexorable decline in lung function and early mortality despite antifibrotic therapy (reviewed in [1,2,3,4]). Median survival remains approximately 2–5 years from diagnosis, with many patients dying from respiratory failure or acute exacerbations [1,5]. Current treatments slow, but do not halt, disease progression and are not curative, underscoring the need to better define upstream drivers and actionable pathways in IPF pathogenesis [6,7].

The prevailing paradigm views IPF as an aberrant wound-healing response to repetitive alveolar epithelial injury in a genetically and environmentally susceptible host (reviewed in [8,9,10]). Alveolar type II (AT2) epithelial cells are central to this model: when intact, they maintain surfactant homeostasis and orchestrate regeneration of the alveolar epithelium; when injured or senescent, they promote maladaptive crosstalk with fibroblasts, immune cells, and the microvasculature [9,10]. Multiple determinants have been implicated in creating a vulnerable epithelial niche—including germline variants in telomere maintenance and mucin genes, ageing, cigarette smoking, and occupational or environmental exposures—but a unifying etiology remains elusive [11,12,13] (reviewed in [8,14]). Telomere biology disorders and shortened leukocyte telomere length, for example, markedly increase risk of pulmonary fibrosis, suggesting that chronic genotoxic or replicative stress primes the distal lung for maladaptive repair [11,15].

Infectious triggers have long been proposed as contributors to this “second hit” model of IPF. A series of observational, mechanistic, and microbiome studies have linked both viruses and bacteria to epithelial injury, acute exacerbations, and worse outcomes (reviewed in [14,16,17]). Herpesviruses, including Epstein-Barr virus (EBV), are frequently detected in IPF lungs ([11,18,19,20,21,22,23,24,25] and reviewed in [21,26]), and can induce endoplasmic reticulum stress, apoptosis, and profibrotic signalling in alveolar epithelial cells [20,27,28]. Parallel work in animal models shows that gammaherpesvirus infection can initiate or amplify fibrotic remodelling in the presence of other injurious stimuli, consistent with a role as a context-dependent cofactor rather than a sole cause (reviewed in [14]).

EBV, a ubiquitous gammaherpesvirus with lifelong latency in B cells and capacity for epithelial tropism, has been repeatedly associated with IPF. Several studies have detected EBV DNA, latent membrane protein 1 (LMP1), or other viral markers within alveolar epithelium, particularly in regions of dense fibrosis and fibroblastic foci [18,22,23,24,28,29,30]. Meta-analytic and narrative reviews conclude that herpesviruses, and EBV in particular, are over-represented in IPF lungs compared with controls and may accelerate disease progression or acute exacerbations, although results are not entirely uniform across cohorts (reviewed in [14,16,31]). Mechanistic studies show that the EBV latent oncoprotein LMP1 activates oncogenic signalling pathways in epithelial cells and fibroblasts, including NF-κB and PI3K, and can cooperate with transforming growth factor-β1 (TGF-β1) to drive epithelial–mesenchymal transition (EMT) [30,32,33]. In primary alveolar epithelial cells, EBV infection upregulates TGF-β1 and engages CUX1/Wnt-related profibrotic programs, providing a plausible link between viral persistence and fibroblast activation [28].

Despite this body of work, several critical uncertainties remain. Tissue-based detection of EBV does not distinguish between latent, abortive, or low-level lytic infection and is subject to sampling bias and assay sensitivity ([34] and reviewed in [14]). Serological investigations in IPF have largely focused on EBV nuclear antigen 1 (EBNA1) and viral capsid antigen (VCA), which may not capture the full complexity of the EBV-specific humoral repertoire [21]. Broader EBV antigenic profiling in other EBV-associated diseases similarly shows that responses to EBNA1 and VCA represent only a small fraction of the total viral proteome [35]. An additional possibility is that some individuals mount only a weak or limited EBV-specific response, consistent with an intrinsic defect in local or systemic EBV immunity. It is unknown whether EBV-specific antibody patterns in IPF are dominated by a narrow set of antigens—consistent with *original antigenic sin*, a phenomenon in which the immune system preferentially recalls antibody responses generated during the initial EBV infection rather than mounting new responses to subsequent antigen exposures (reviewed in [36,37])—or whether they reflect broad enrichment across lytic and structural proteins, indicative of chronic, low-grade viral activity (reviewed in [38,39]). Observations from EBV-associated neurological disease, especially multiple sclerosis (MS), show that fine antigenic specificity carries mechanistic and prognostic information (reviewed in [35]), suggesting that dissecting antigen-resolved EBV serology in IPF may offer similar insights.

To address these questions, we used a proteome-wide EBV protein microarray to map IgG responses to latent, lytic, tegument, and structural antigens in IPF and controls. We asked whether the EBV-specific serological repertoire in IPF is characterized by antigen-focused dominance or by broad-based elevation across the viral proteome, and whether enriched antigens correspond to proteins with established epithelial- or fibroblast-modulating functions.

## 2. Results

### 2.1. Study Cohorts

A total of 47 individuals were included in the study (IPF: *n* = 32; Controls: *n* = 15) (Table 1). There was no significant difference in mean age between IPF and control cohorts (*p* = 0.328, Welch’s *t*-test). The groups were not balanced by sex, with the IPF cohort comprising a significantly higher proportion of males (87.5%; *p* = 0.001, Fisher’s exact test), consistent with the recognized male predominance of IPF [40]. 

### 2.2. Proteome-Wide Patterns at the Individual Level

**Figure 1:**
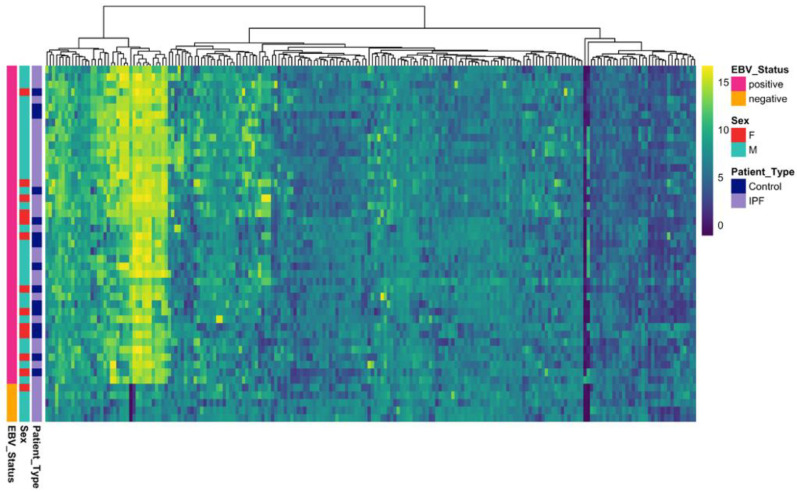
Heatmap showing Epstein–Barr virus (EBV) proteome reactivity across study participants. Rows represent individual subjects (IPF patients (*n* = 32) and healthy controls (*n* = 15)), and columns represent EBV antigens. Color intensity indicates signal strength normalized using variance-stabilizing normalization (VSN).

An increased EBV-specific humoral response was observed in the IPF group compared with controls, with a broad spectrum of antigen reactivity evident across IPF cases (Figure 1). Notably, a discrete subset of IPF cases (*n* = 5, 16%) was seronegative for EBV. All five cases were confirmed EBV-seronegative by diagnostic serology at Pathology Queensland; these samples were excluded from all downstream antibody analyses. Negative-control features were also significantly lower in IPF relative to controls in females (Welch *p* = 0.0078), indicating baseline differences in assay signal and supporting inclusion of sex as a covariate in downstream statistical models.

### 2.3. Global EBV-Directed Humoral Activity 

**Figure 2:**
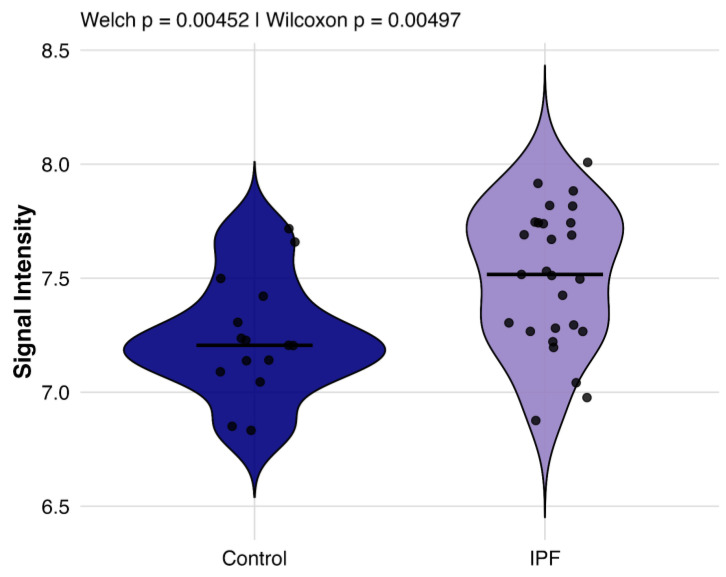
Comparison of global EBV antibody responses between individuals with IPF and healthy controls. Violin plots display the distribution of per-subject mean VSN signal intensity across all EBV proteins for EBV+ IPF patients (purple, *n* = 27) and healthy controls (blue, *n* = 15).

We quantified the mean EBV IgG signal intensity per participant across all EBV proteins on a variance-stabilization normalization (VSN) scale (Figure 2). IPF patients exhibited a higher global reactivity relative to controls (Welch’s *t*-test *p* = 0.005), which remained significant after adjustment for sex in a linear model (*p* = 0.012). These data indicate a proteome-wide elevation of EBV-directed humoral activity in IPF.

Given the marked sex imbalance between IPF and control cohorts, additional sex-specific sensitivity analyses were performed, restricted to EBV-positive individuals only. In a multivariable linear regression model including disease status, sex, age, and a disease-by-sex interaction term, IPF status remained significantly associated with higher global EBV IgG levels (β = 0.56, *p* = 0.0067). Neither sex (*p* = 0.53) nor age (*p* = 0.45) was independently associated with global EBV IgG, and the disease-by-sex interaction term did not reach statistical significance (*p* = 0.10), indicating no strong evidence that the IPF effect differed by sex.

In sex-stratified analyses, global EBV IgG levels were higher in IPF than in controls among male EBV-positive participants (mean difference = 0.17), although this difference did not reach statistical significance (*p* = 0.28), likely reflecting limited power due to the small number of male controls (*n* = 6). Among female EBV-positive participants, a larger difference was observed (mean difference = 0.55, *p* = 0.014); however, this subgroup was particularly underpowered (IPF *n* = 3), and these results should be interpreted with caution.

### 2.4. Differential Antibody Responses in IPF Compared with Healthy Controls

**Figure 3:**
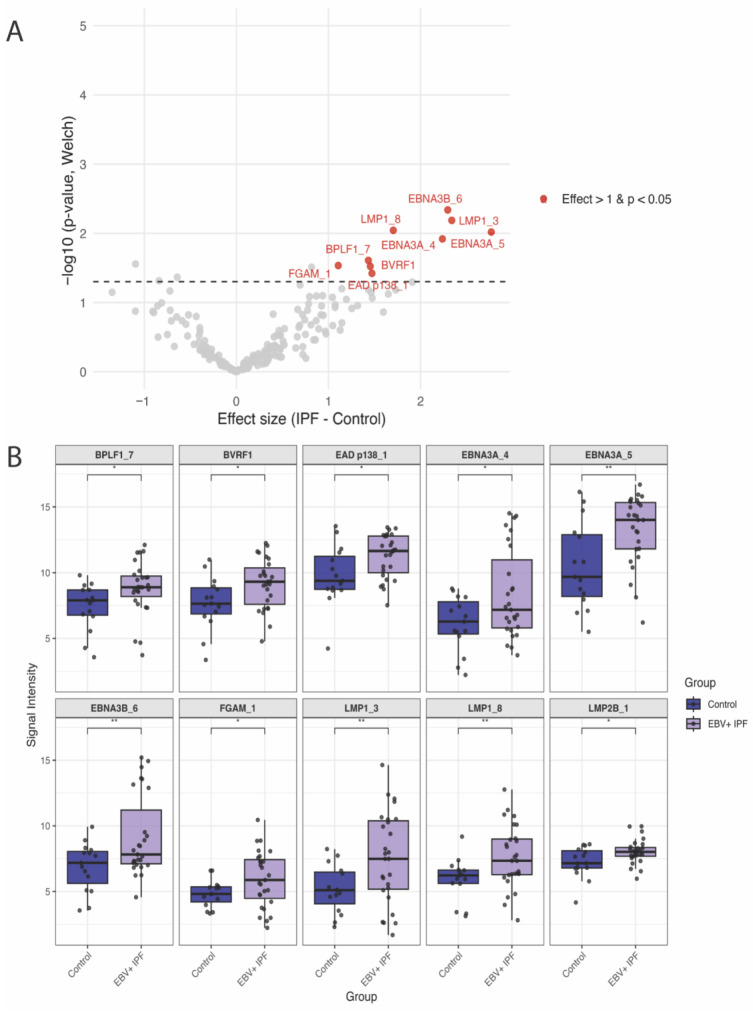
Differential EBV-specific IgG responses in EBV+ IPF patients (*n* = 27) versus healthy controls (*n* = 15). (**A**). Volcano plot showing proteome-wide differences in EBV-specific IgG antibody responses between EBV+ IPF patients and controls. Each point represents one EBV protein (total *n* = 202). The *x*-axis shows the effect size (difference in VSN-normalized signal intensity: IPF–Control), and the *y*-axis shows –log_10_(*p*) from Welch’s *t*-test. Horizontal dashed line indicates the statistical threshold (*p* = 0.05). (**B**). Boxplots of the 10 differentially elevated antigens in EBV+ IPF patients relative to controls by Welch’s t-tests (nominal *p* value < 0.05).

In whole proteome-wide analyses, no individual antigen reached false discovery rate (FDR) significance using either Welch’s *t*-tests or sex-adjusted linear regression models. Ten antigens showed elevated IgG responses in IPF compared with controls at the nominal level (*p* < 0.05), and are therefore considered exploratory (Figure 3, Table 2 and Table S1). These included EBV latent antigens (EBNA3A, EBNA3B, LMP1, LMP2B) and late lytic antigens (BVRF1, BPLF1), as well as an uncharacterized antigen (FGAM) and one immediate early/early (IE/E) lytic antigen (EAD p138 [BALF2]) (Table 2 and Table S1). Of these, two antigens (EAD p138 [BALF2] and LMP2B) remained nominally significant after sex adjustment, while an additional five antigens (2 × LMP1, 2 × VCAp160 [BcLF1], and BVRF2) were nominally significant only in sex-adjusted linear regression models (Table 2 and Table S1).

## 3. Discussion

In this study, we applied proteome-wide serological profiling to investigate EBV-specific antibody responses in individuals with idiopathic pulmonary fibrosis (IPF) compared with disease-free healthy controls. Our findings provide new evidence for a broad elevation of EBV-directed humoral activity in IPF, consistent with an altered host–virus immune balance and low-grade systemic immune engagement rather than a direct causal role for EBV in disease pathogenesis.

Global EBV IgG reactivity was significantly increased in IPF patients compared to controls, even after adjusting for sex differences. This pattern is consistent with the concept of a systemic, non-antigen-specific amplification of EBV immune responses, rather than selective targeting of a few dominant antigens. 

Importantly, additional sensitivity analyses indicate that this global elevation of EBV-directed humoral activity is not solely attributable to the marked sex imbalance between cohorts. When analyses were restricted to EBV-positive individuals and examined within sex strata, the direction of effect remained consistent with higher global EBV IgG levels in IPF. Although statistical significance was attenuated in male-only analyses, likely reflecting limited power due to the small number of male controls, the absence of a significant disease-by-sex interaction suggests that sex does not strongly modify the association between IPF and elevated EBV IgG. Together, these findings support the robustness of the global EBV IgG signal in IPF while highlighting the constraints imposed by sample size on definitive sex-specific inference.

At the individual antigen level, no antigen remained significant after FDR correction, although several latent (EBNA3A, EBNA3B, LMP1, LMP2B) and late lytic proteins (BVRF1, BPLF1, VCAp160) demonstrated nominally elevated reactivity in IPF patients compared to controls. 

EBV has long been implicated as a potential cofactor in IPF, with multiple studies demonstrating EBV DNA, EBV RNA, or latent proteins within alveolar epithelial cells. However, most prior studies have focused on the presence or absence of viral markers rather than the host immune response. Our findings add a serological dimension to prior tissue-based and mechanistic studies implicating EBV in IPF. Earlier work focused on the presence or absence of viral nucleic acids or LMP1 staining in lung biopsies and bronchoalveolar lavage [18,22,23,24,28,29,30]. Multiple narrative and systematic assessments emphasize that herpesvirus activity (including EBV) has been variably detected in IPF lungs with inconsistent prevalence across cohorts ([11] and reviewed in [14,16,31]).

The recurrent appearance of latency-associated oncoproteins and transcriptional regulators (LMP1, LMP2B, EBNA3 family) is notable given their established roles in viral persistence, immune modulation, and cellular transformation (reviewed in [36,37]). LMP1 acts as a viral mimic of CD40 signaling [32], driving NF-κB activation, cytokine release, and fibroblast proliferation—pathways directly relevant to fibrotic remodeling [33]. Elevated anti–LMP2B likely reflects episodic EBV reactivation; LMP2B lowers the threshold for lytic induction in human B cells [38]. EBV reactivation has also been documented in the IPF lung epithelium, where it co-localizes with epithelial ER stress, a key driver of fibrosis [20], while EBNA3 proteins regulate host and viral gene expression, contributing to immune evasion and long-term persistence of infected cells (reviewed in [36]). The prominence of these responses suggests that chronic antigenic drive from latent EBV reservoirs may underpin persistent immune activation in IPF.

In parallel, recognition of replication-associated antigens such as EAD p138 [BALF2], a DNA-binding protein essential for viral DNA replication during the lytic cycle, and late structural and tegument proteins, including VCAp160 [BcLF1], BVRF1, BVRF2, and BPLF1, indicates immune engagement with intermittent lytic replication. These proteins mediate essential processes such as viral DNA synthesis, capsid assembly, and epithelial entry, and their detection implies cycles of viral reactivation in IPF lung tissue [39,41]. Importantly, BPLF1 is a multifunctional tegument protein with deubiquitinase activity that enhances viral infectivity and disrupts host immune pathways, further supporting a model in which EBV exploits immune evasion to sustain long-term infection [42,43].

Taken together, the diffuse pattern of elevated antibody responses across both latent and lytic EBV proteins suggests that the humoral immune responses observed in IPF are unlikely to be random. Rather, it reflects low-grade, systemic immune engagement with both latent and replicative viral reservoirs. These findings reinforce the concept of “smoldering EBV activity” as a potential pathogenic feature in IPF, in which persistent latency and intermittent reactivation create a chronic antigenic milieu associated with epithelial injury, immune activation, and fibrotic remodelling. 

The identification of a confirmed EBV-seronegative subgroup (16%) within IPF is notable. No recent Australia-wide seropositivity data are available; however, an earlier Western Australia study reported ~92% EBV seropositivity in young adults [44], while a recent meta-analysis of global EBV sero-epidemiology found that seroprevalence rises from ~66% in early childhood to >95% in adults, approaching ~99–100% by about age 30 [45] which is consistent with the accepted prevalence of EBV seropositivity in adults worldwide. The presence of an apparently higher proportion of EBV-seronegative individuals in this IPF cohort should be interpreted cautiously, given the small sample size and potential alternative explanations, including assay sensitivity, temporal variation in serostatus, or host immune differences. While this observation raises the possibility of etiologic heterogeneity—whereby EBV may act as a cofactor in a subset of IPF cases while EBV-independent pathways may predominate in others—definitive conclusions will require validation in larger, independent cohorts. Whether EBV status alters clinical course or response to therapy warrants prospective evaluation.

Our results align with reports of EBV replication in alveolar epithelial cells [18] and observational associations between viral infection and IPF [21]. This hypothesis is further supported by a progression of clinical data; an initial open-label pilot study of ganciclovir showed a clinical response in a subset of patients [46], which provided the rationale for a subsequent Phase I randomized trial of valganciclovir. This more formal trial confirmed the drug’s safety and, while not powered for efficacy, demonstrated a durable trend toward benefit that was sustained for over 12 months [47]. 

Parallels with multiple sclerosis (MS) highlight the importance of antigen-resolved EBV immunity. MS is characterized by highly selective, high-affinity responses to EBNA1 and EBNA3 family peptides, with strong temporal and mechanistic links to disease onset (reviewed in [35]). Although IPF lacks the autoimmune features of MS, and antibodies in MS are largely detected within the cerebrospinal fluid, both conditions may involve persistent EBV activity within a vulnerable tissue niche, generating chronic low-grade injury or stress signalling. In IPF, our findings suggest this may occur through episodic epithelial reactivation rather than B-cell-centred autoimmunity.

These observations raise potential translational opportunities. Antigen-resolved EBV serology could act as a non-invasive biomarker for epithelial viral activity. Identified antigens with known epithelial-injury or profibrotic functions offer a shortlist for mechanistic testing in AT2 cells, fibroblasts, organoids, or precision-cut lung slices. Larger cohorts will be required to test whether specific EBV serological signatures associate with accelerated decline, exacerbation risk, or therapeutic response.

Methodologically, in our study, the observation of lower negative-control signals in IPF (particularly among females) highlights the importance of covariate adjustment and careful normalization in serological proteomics studies. We acknowledge several limitations. The overall sample size was modest (*n* = 47), which limits statistical power for probe-level analyses after multiple-testing correction and may have contributed to the absence of FDR-significant individual antigens despite consistent effect directions. There was also a marked sex imbalance between IPF and control groups, reflecting the known male predominance of IPF; sex was therefore included as a covariate in all global and probe-wise linear regression analyses, and additional sex-specific sensitivity analyses restricted to EBV-positive individuals were performed. However, the modest sample size, particularly within sex-stratified subgroups, limits statistical power, and residual sex-related effects cannot be fully excluded. In addition, baseline covariates beyond age and sex, including smoking status, medication use, and other potential confounders, were not collected or available for this cohort; therefore, comparability between IPF and control groups beyond age and sex could not be assessed.

While the reproducibility and technical robustness of the EBV protein microarray platform, variance-stabilizing normalization (VSN), and analytical pipeline have been established in multiple prior large-scale sero-epidemiological and disease-association studies using the same array design, all samples in the present study were processed using standardized protocols with internal controls and uniform scanner settings. Finally, the use of recombinant proteins may not fully capture conformational epitopes present in native viral proteins. Independent cohort replication will be required to confirm biological generalizability.

Collectively, these data support a working model wherein smoldering EBV activation is associated with repetitive epithelial injury and maladaptive repair in IPF, manifesting as diffuse serological elevation rather than antigen-specific dominance. Larger studies integrating lung-tissue viromics, longitudinal serology, viral load, and clinical phenotyping are needed to validate these observations and to determine their translational implications.

## 4. Materials and Methods

### 4.1. Study Population 

Plasma was obtained from 32 patients with IPF and 15 healthy disease-free controls under the Prince Charles Hospital Human Research Ethics Committee (HREC) approval (HREC/2018/QPCH/44293) with written informed consent. Demographic imbalances (notably sex) were accounted for in downstream analyses. Analysis of these samples by protein microarray was conducted upon approval by HREC of The University of Queensland (2024/HE000886 approved 19 April 2024).

### 4.2. Plasma Isolation, Storage, and Transport

Blood was collected in 6 mL Lithium Heparin Vacutainer tubes (Becton, Dickinson and Company, Plymouth, Devon, UK) and centrifuged at 1500× *g* for 10 min to separate the plasma. Plasma was divided into 1 mL aliquots and frozen at −80 °C at the Prince Charles Hospital (Brisbane, Queensland (QLD), Australia). Vials were transported on dry ice by private vehicle to the University of Queensland and stored at −80 °C until use.

### 4.3. Custom EBV Protein Microarrays

Custom EBV protein microarrays contained the entire EBV proteome of 86 proteins, designed using sequences available on GenBank from five strains of EBV (AG876, Akata, B95-8, Mutu, Raji) and splice variants [48,49,50]. In total, 202 EBV proteins were printed on the arrays (Antigen Discovery Inc., Irvine, CA, USA). Each array contained probing anti-IgG controls, noDNA (no translated protein) controls, buffer controls, and mock clones. Three synthetic EBV peptides used as positive controls were part of the 202 EBV proteins, comprising viral capsid antigen (VCA), EBV nuclear antigen 1 (EBNA1), and EBV early antigen (EAD1). These three EBV proteins are used in clinical testing and are biomarkers for EBV serology.

Slides were stored at 4 °C upon arrival and moved to a room temperature desiccator cabinet several days before probing. On the day of probing commencement, each microarray was rehydrated with protein-free array blocking buffer (SurModics, Inc., Eden Prairie, MN, USA). Plasma samples were diluted at 1:100 in 20% *E. coli* lysate and blocking buffer solution and incubated for 30 min at room temperature to block anti-*E. coli* antibodies. Each sample was added to the corresponding pad in the microarray and placed on a slow rocker overnight at 4 °C. Plasma samples were removed the following day, and the microarray was washed with Tris-buffered saline with Tween20 (TTBS). Biotin-conjugated goat anti-human IgG (1:1000 dilution) (Jackson ImmunoResearch Laboratories Inc., West Grove, PA, USA) was added and incubated for one hour at room temperature. Anti-human IgG was then removed, the microarray was washed with TTBS, and streptavidin-conjugated Surelight^®^ P3 (Columbia Biosciences, Frederick, MD, USA) was added at a 1:200 dilution in blocking buffer and incubated in the dark for one hour at room temperature. The streptavidin substrate was removed, the microarray was washed with TTBS, Tris-buffered saline (TBS), and finally sterile water before being centrifuged and placed in a light-proof box in a desiccator cabinet to dry overnight.

### 4.4. Data Acquisition & Analysis 

Air-dried probed microarray slides were scanned using the Axon GenePix 4300A microarray scanner (Molecular Devices, San Jose, CA, USA) using a 635 nm excitation wavelength with laser power at 100%. The photomultiplier tube (PMT) values were adjusted to maximize signal intensity without over-saturating features, and a constant PMT of 350 was used for each slide. Signals were quantified using Axon GenePix Pro 7 image analysis software (v7.4, Molecular Devices, San Jose, CA, USA), with a GenePix Array List file used to define feature location and identity. 

The raw data signal fluorescence intensities were corrected for all protein spots by subtracting individual-specific background from the mean of the four noDNA negative controls. Background-corrected data were normalized using variance-stabilizing normalization (VSN) using the VSN Bioconductor package in RStudio (v4.3, Posit, Boston, MA, USA). Global antibody responses were evaluated by Welch tests on per-sample means and by multivariable linear models adjusting for sex. To further assess the impact of sex imbalance and potential residual confounding, additional sex-specific sensitivity analyses were performed. These analyses were restricted to EBV-positive individuals only. A global EBV IgG score was calculated for each participant as the mean signal across all EBV probes following background correction and variance-stabilizing normalization. Multivariable linear regression models included disease status (IPF vs. control), sex, age, and a disease-by-sex interaction term. In addition, sex-stratified comparisons (male and female separately) were conducted to evaluate whether IPF-associated differences in global EBV IgG persisted within each sex.

Probe-wise comparisons used Welch’s *t*-tests and linear models. In addition to reporting nominal *p*-values from statistical tests, the Benjamini and Hochberg false discovery rate (FDR = 5%) method was applied to account for multiple tests. All analyses were conducted in RStudio (v4.3, Posit, Boston, MA, USA). 

For probe-level analyses, effect sizes were summarized as differences in group means on the VSN-normalized scale (IPF—Control). Uncertainty in group mean estimates and mean differences was quantified using non-parametric bootstrap 95% confidence intervals (2000 resamples), generated by resampling individuals with replacement within each group. This approach was chosen to minimize reliance on distributional assumptions given the skewed nature of antibody intensity data and the modest sample size.

## 5. Conclusions

IPF is associated with a diffuse elevation of EBV-directed antibody responses that is robust to sex adjustment, with no single antigen meeting FDR significance. However, elevated anti-EBV responses were observed for 10 EBV proteins, supporting the concept that IPF is associated with a broad elevation of EBV-directed humoral responses. A validated EBV-negative subgroup underscores disease heterogeneity. These findings support a model of smoldering viral activation and chronic humoral response associated with IPF in a subset of patients.

## Figures and Tables

**Table 1:** Demographic and EBV serostatus characteristics of the study cohorts.

Group	N	Male (*n*, %)	Age (Mean ± SD)	EBV+ (*n*, %)
IPF	32	28, 87.5%	60.9 ± 8.3	27, 84%
Control	15	6, 40%	57.9 ± 9.9	15, 100%

**Table 2:** Differential EBV-specific IgG responses in IPF. Mean VSN signal intensities and confidence intervals (CI) for controls (*n* = 15) and EBV+ IPF patients (*n* = 27), effect sizes (IPF–Control), Welch *p*-values, and FDR-adjusted *p*-values from sex-adjusted models. IE/E—immediate early/early.

Marker	Mean Control (CI)	Mean IPF (CI)	Effect	*p*-Value (Welch)	FDR	*p*-Value (lin. reg.)	Life Cycle
EAD p138_1/BALF2	9.79 (8.69–10.91)	11.26 (10.65–11.84)	1.47	**0.038**	0.645	0.005 *	IE/E lytic
LMP2B_1	7.22 (6.57–7.75)	8.04 (7.70–8.39)	0.82	**0.031**	0.622	0.025 *	Latent
EBNA3B_6	6.87 (5.95–7.75)	9.17 (8.06–10.34)	2.30	**0.005**	0.484	0.123	Latent
LMP1_3	5.18 (4.34–6.06)	7.52 (6.20–8.84)	2.34	**0.006**	0.484	0.174	Latent
LMP1_8	5.92 (5.13–6.66)	7.63 (6.71–8.49)	1.70	**0.009**	0.484	0.121	Latent
EBNA3A_5	10.50 (8.86–12.11)	13.27 (12.26–14.17)	2.77	**0.010**	0.484	0.198	Latent
EBNA3A_4	6.10 (5.09–7.09)	8.34 (7.14–9.63)	2.24	**0.012**	0.487	0.132	Latent
BPLF1_7	7.40 (6.52–8.20)	8.83 (8.05–9.59)	1.43	**0.025**	0.622	0.062	Late lytic
FGAM_1	4.85 (4.36–5.32)	5.95 (5.19–6.78)	1.11	**0.029**	0.622	0.102	Unknown
BVRF1	7.69 (6.67–8.65)	9.15 (8.42–9.81)	1.45	**0.030**	0.622	0.059	Late lytic
VCAp160_3/BcLF1	7.66 (6.59–8.73)	8.98 (7.89–10.05)	1.32	0.110	0.645	0.025 *	Late lytic
LMP1_1	6.39 (5.22–7.48)	7.64 (6.72–8.56)	1.25	0.122	0.645	0.027 *	Latent
VCAp160_4/BcLF1	8.24 (7.48–9.06)	8.99 (7.94–10.08)	0.75	0.289	0.816	0.044 *	Late lytic
BVRF2_2	6.15 (5.13–7.14)	6.96 (6.30–7.61)	0.81	0.210	0.737	0.049 *	Late lytic
LMP1_10	8.08 (6.88–9.05)	9.15 (8.56–9.70)	1.07	0.108	0.645	0.012 *	Latent

Antigens showing nominal (unadjusted) significance in IgG responses between IPF patients and controls, as determined by Welch’s *t*-tests or linear regression, are highlighted in bold (*p* < 0.05). * The antigens remained nominally significant after sex adjustment in linear regression models.

## Data Availability

The data that support the findings of this study are available from the corresponding author upon reasonable request. The data are not publicly available due to privacy and ethical restrictions.

## Supplementary Materials

The following supporting information can be downloaded at https://www.mdpi.com/article/10.3390/ijms27020783/s1.

## Abbreviations

The following abbreviations are used in this manuscript:

IPF	Idiopathic Pulmonary Fibrosis
EBV	Epstein–Barr Virus
FDR	False discovery rate
AT2	Alveolar type II
LMP	Latent membrane protein
NF-κB	Nuclear factor kappa B
PI3K	Phosphoinositide 3-kinase
TGF-β1	Transforming growth factor beta 1
EMT	Epithelial-mesenchymal transition
EBNA	EBV nuclear antigen
VCA	Viral capsid antigen
MS	Multiple sclerosis
VSN	Variance-stabilizing normalization
IE/E	Immediate early/early
CI	Confidence interval
HREC	Human research ethics committee
BD	Becton, Dickinson and Company
EAD	EBV early antigen
TTBS	Tris-buffered saline with Tween20
TBS	Tris-buffered saline
PMT	Photomultiplier tube

## References

[1] Lederer D.J., Martinez F.J. (2018). Idiopathic Pulmonary Fibrosis. N. Engl. J. Med..

[2] Glass D.S., Grossfeld D., Renna H.A., Agarwala P., Spiegler P., Kasselman L.J., Glass A.D., DeLeon J., Reiss A.B. (2020). Idiopathic pulmonary fibrosis: Molecular mechanisms and potential treatment approaches. Respir. Investig..

[3] King T.E., Pardo A., Selman M. (2011). Idiopathic pulmonary fibrosis. Lancet.

[4] Richeldi L., Collard H.R., Jones M.G. (2017). Idiopathic pulmonary fibrosis. Lancet.

[5] Ley B., Collard H.R., King T.E. (2011). Clinical course and prediction of survival in idiopathic pulmonary fibrosis. Am. J. Respir. Crit. Care Med..

[6] Noor S., Nawaz S., Chaudhuri N. (2021). Real-World Study Analysing Progression and Survival of Patients with Idiopathic Pulmonary Fibrosis with Preserved Lung Function on Antifibrotic Treatment. Adv. Ther..

[7] Kou M., Jiao Y., Li Z., Wei B., Li Y., Cai Y., Wei W. (2024). Real-world safety and effectiveness of pirfenidone and nintedanib in the treatment of idiopathic pulmonary fibrosis: A systematic review and meta-analysis. Eur. J. Clin. Pharmacol..

[8] Maher T.M., Wells A.U., Laurent G.J. (2007). Idiopathic pulmonary fibrosis: Multiple causes and multiple mechanisms?. Eur. Respir. J..

[9] Camelo A., Dunmore R., Sleeman M.A., Clarke D.L. (2014). The epithelium in idiopathic pulmonary fibrosis: Breaking the barrier. Front. Pharmacol..

[10] Confalonieri P., Volpe M.C., Jacob J., Maiocchi S., Salton F., Ruaro B., Confalonieri M., Braga L. (2022). Regeneration or Repair? The Role of Alveolar Epithelial Cells in the Pathogenesis of Idiopathic Pulmonary Fibrosis (IPF). Cells.

[11] Kropski J.A., Pritchett J.M., Zoz D.F., Crossno P.F., Markin C., Garnett E.T., Degryse A.L., Mitchell D.B., Polosukhin V.V., Rickman O.B. (2015). Extensive phenotyping of individuals at risk for familial interstitial pneumonia reveals clues to the pathogenesis of interstitial lung disease. Am. J. Respir. Crit. Care Med..

[12] Chin D., Hernandez-Beeftink T., Donoghue L., Guillen-Uio B., Leavy O.C., Adegunsoye A., Booth H.L., Fahy W.A., Fingerlin T.E., CleanUP-IPF Investigators of the Pulmonary Trials Cooperative (2025). Genome-wide association study of Idiopathic Pulmonary Fibrosis susceptibility using clinically-curated European-ancestry datasets. medRxiv.

[13] Allen R.J., Stockwell A., Oldham J.M., Guillen-Guio B., Schwartz D.A., Maher T.M., Flores C., Noth I., Yaspan B.L., Jenkins R.G. (2022). Genome-wide association study across five cohorts identifies five novel loci associated with idiopathic pulmonary fibrosis. Thorax.

[14] Williams K.J. (2014). Gammaherpesviruses and pulmonary fibrosis: Evidence from humans, horses, and rodents. Vet. Pathol..

[15] Armanios M.Y., Chen J.J., Cogan J.D., Alder J.K., Ingersoll R.G., Markin C., Lawson W.E., Xie M., Vulto I., Phillips J.A. (2007). Telomerase mutations in families with idiopathic pulmonary fibrosis. N. Engl. J. Med..

[16] Molyneaux P.L., Maher T.M. (2013). The role of infection in the pathogenesis of idiopathic pulmonary fibrosis. Eur. Respir. Rev..

[17] Molyneaux P.L., Maher T.M. (2014). Respiratory microbiome in IPF: Cause, effect, or biomarker?. Lancet Respir. Med..

[18] Egan J.J., Stewart J.P., Hasleton P.S., Arrand J.R., Carroll K.B., Woodcock A.A. (1995). Epstein-Barr virus replication within pulmonary epithelial cells in cryptogenic fibrosing alveolitis. Thorax.

[19] Kelly B.G., Lok S.S., Hasleton P.S., Egan J.J., Stewart J.P. (2002). A rearranged form of Epstein-Barr virus DNA is associated with idiopathic pulmonary fibrosis. Am. J. Respir. Crit. Care Med..

[20] Lawson W.E., Crossno P.F., Polosukhin V.V., Roldan J., Cheng D.S., Lane K.B., Blackwell T.R., Xu C., Markin C., Ware L.B. (2008). Endoplasmic reticulum stress in alveolar epithelial cells is prominent in idiopathic pulmonary fibrosis: Association with altered surfactant protein processing and herpesvirus infection. Am. J. Physiol.—Lung Cell. Mol. Physiol..

[21] Sheng G., Chen P., Wei Y., Yue H., Chu J., Zhao J., Wang Y., Zhang W., Zhang H.L. (2020). Viral infection increases the risk of idiopathic pulmonary fibrosis: A meta-analysis. Chest.

[22] Stewart J.P., Egan J.J., Ross A.J., Kelly B.G., Lok S.S., Hasleton P.S., Woodcock A.A. (1999). The detection of Epstein-Barr virus DNA in lung tissue from patients with idiopathic pulmonary fibrosis. Am. J. Respir. Crit. Care Med..

[23] Tang Y.W., Johnson J.E., Browning P.J., Cruz-Gervis R.A., Davis A., Graham B.S., Brigham K.L., Oates J.A., Loyd J.E., Stecenko A.A. (2003). Herpesvirus DNA is consistently detected in lungs of patients with idiopathic pulmonary fibrosis. J. Clin. Microbiol..

[24] Tsukamoto K., Hayakawa H., Sato A., Chida K., Nakamura H., Miura K. (2000). Involvement of Epstein-Barr virus latent membrane protein 1 in disease progression in patients with idiopathic pulmonary fibrosis. Thorax.

[25] Vergnon J.M., de Thé G., Weynants P., Vincent M., Mornex J.F., Brune J. (1984). Cryptogenic fibrosing alveolitis and Epstein-Barr virus: An association?. Lancet.

[26] Duckworth A., Longhurst H.J., Paxton J.K., Scotton C.J. (2021). The Role of Herpes Viruses in Pulmonary Fibrosis. Front. Med..

[27] Isler J.A., Skalet A.H., Alwine J.C. (2005). Human cytomegalovirus infection activates and regulates the unfolded protein response. J. Virol..

[28] Malizia A.P., Keating D.T., Smith S.M., Walls D., Doran P.P., Egan J.J. (2008). Alveolar epithelial cell injury with Epstein-Barr virus upregulates TGFβ1 expression. Am. J. Physiol. Lung Cell Mol. Physiol..

[29] Magro C.M., Allen J., Pope-Harman A., Waldman W.J., Moh P., Rothrauff S., Ross P. (2003). The role of microvascular injury in the evolution of idiopathic pulmonary fibrosis. Am. J. Clin. Pathol..

[30] Sides M.D., Klingsberg R.C., Shan B., Gordon K.A., Nguyen H.T., Lin Z., Takahashi T., Flemington E.K., Lasky J.A. (2011). The Epstein-Barr virus latent membrane protein 1 and transforming growth factor-β1 synergistically induce epithelial-mesenchymal transition in lung epithelial cells. Am. J. Respir. Cell Mol. Biol..

[31] Jafarian A.H., Roshan N.M., Ayatollahi H., Omidi A.A., Ghaznavi M., Gharib M. (2020). Epstein-Barr Virus and Human Herpesvirus 8 in Idiopathic Pulmonary Fibrosis. Iran. J. Pathol..

[32] Lam N., Sugden B. (2003). CD40 and its viral mimic, LMP1: Similar means to different ends. Cell. Signal..

[33] Mainou B.A., Everly D.N., Raab-Traub N. (2005). Epstein-Barr virus latent membrane protein 1 CTAR1 mediates rodent and human fibroblast transformation through activation of PI3K. Oncogene.

[34] Dos Santos G.C., Parra E.R., Stegun F.W., Cirqueira C.S., Capelozzi V.L. (2013). Immunohistochemical detection of virus through its nuclear cytopathic effect in idiopathic interstitial pneumonia other than acute exacerbation. Braz. J. Med. Biol. Res..

[35] Comabella M., Kakalacheva K., Rio J., Munz C., Montalban X., Lunemann J.D. (2012). EBV-specific immune responses in patients with multiple sclerosis responding to IFNβ therapy. Mult. Scler..

[36] Styles C.T., Paschos K., White R.E., Farrell P.J. (2018). The Cooperative Functions of the EBNA3 Proteins Are Central to EBV Persistence and Latency. PLoS Pathog..

[37] Kang M.S., Kieff E. (2015). Epstein–Barr virus latent genes. Exp. Mol. Med..

[38] Rechsteiner M.P., Berger C., Zauner L., Sigrist J.A., Weber M., Longnecker R., Bernasconi M., Nadal D. (2008). Latent membrane protein 2B regulates susceptibility to induction of lytic Epstein–Barr virus infection. J. Virol..

[39] Xiao J., Palefsky J.M., Herrera R., Berline J., Tugizov S.M. (2008). The Epstein-Barr virus BMRF-2 protein facilitates virus attachment to oral epithelial cells. Virology.

[40] Sia L.C., Amanda G., Bączek K., Achaiah A., Sesé L., Chaudhuri N. (2025). Gender differences in clinical features, comorbidities and prognostic outcomes in idiopathic pulmonary fibrosis—A retrospective cohort analysis from the British Thoracic Society Interstitial Lung Disease Registry. BMJ Open.

[41] Murayama K., Nakayama S., Kato-Murayama M., Akasaka R., Ohbayashi N., Kamewari-Hayami Y., Terada T., Shirouzu M., Tsurumi T., Yokoyama S. (2009). Crystal structure of epstein-barr virus DNA polymerase processivity factor BMRF1. J. Biol. Chem..

[42] Whitehurst C.B., Vaziri C., Shackelford J., Pagano J.S. (2012). Epstein-Barr virus BPLF1 deubiquitinates PCNA and attenuates polymerase η recruitment to DNA damage sites. J. Virol..

[43] van Gent M., Braem S.G., de Jong A., Delagic N., Peeters J.G., Boer I.G., Moynagh P.N., Kremmer E., Wiertz E.J., Ovaa H. (2014). Epstein-Barr virus large tegument protein BPLF1 contributes to innate immune evasion through interference with toll-like receptor signaling. PLoS Pathog..

[44] Lai P.K., Mackay-Scollay E.M., Alpers M.P. (1975). Epidemiological studies of Epstein-Barr herpesvirus infection in Western Australia. J. Hyg..

[45] Muckian M.D., Shi T., Qarkaxhija V., Kapoor S., Morgan T., Stagg H.R. (2025). Equity in protection: Bridging global data gaps for an EBV vaccine-a systematic review and meta-analysis. BMJ Glob. Health.

[46] Egan J.J., Adamali H.I., Lok S.S., Stewart J.P., Woodcock A.A. (2011). Ganciclovir antiviral therapy in advanced idiopathic pulmonary fibrosis: An open pilot study. Pulm. Med..

[47] Blackwell T.S., Hewlett J.C., Mason W.R., Martin S., Del Greco J., Ding G., Wu P., Lancaster L.H., Loyd J.E., Dudenhofer R.B. (2021). A Phase I Randomized, Controlled, Clinical Trial of Valganciclovir in Idiopathic Pulmonary Fibrosis. Ann. Am. Thorac. Soc..

[48] Coghill A.E., Pfeiffer R.M., Proietti C., Hsu W.L., Chien Y.C., Lekieffre L., Krause L., Teng A., Pablo J., Yu K.J. (2018). Identification of a Novel, EBV-based Antibody Risk Stratification Signature for Early Detection of Nasopharyngeal Carcinoma in Taiwan. Clin. Cancer Res..

[49] Liu Z., Jarrett R.F., Hjalgrim H., Proietti C., Chang E.T., Smedby K.E., Yu K.J., Lake A., Troy S., McAulay K.A. (2020). Evaluation of the antibody response to the EBV proteome in EBV-associated classical Hodgkin lymphoma. Int. J. Cancer.

[50] Liu Z., Sarathkumara Y.D., Chan J.K.C., Kwong Y.L., Lam T.H., Ip D.K.M., Chiu B.C., Xu J., Su Y.C., Proietti C. (2021). Characterization of the humoral immune response to the EBV proteome in extranodal NK/T-cell lymphoma. Sci. Rep..

